# Multiple biological functions of Twist1 in various cancers

**DOI:** 10.18632/oncotarget.14608

**Published:** 2017-01-12

**Authors:** Zhixiang Zhao, Mohammad Aminur Rahman, Zhuo G. Chen, Dong M. Shin

**Affiliations:** ^1^ Department of Hematology and Medical Oncology, Winship Cancer Institute of Emory University, Atlanta, GA, United States of America; ^2^ Department of Dermatology, Xiangya Hospital, Central South University, Changsha, Hunan, China

**Keywords:** twist1, EMT, stemness, angiogenesis, chemo-resistance

## Abstract

Twist1 is a well-known regulator of transcription during embryonic organogenesis in many species. In humans, Twist1 malfunction was first linked to Saethre-Chotzen syndrome and later identified to play an essential role in tumor initiation, stemness, angiogenesis, invasion, metastasis, and chemo-resistance in a variety of carcinomas, sarcomas, and hematological malignances. In this review, we will first focus on systematically elaborating the diverse pathological functions of Twist1 in various cancers, then delineating the intricate underlying network of molecular mechanisms, based on which we will summarize current therapeutic strategies in cancer treatment that target and modulate Twist1-involved signaling pathways. Most importantly, we will put special emphasis on revealing the independence and interdependency of these multiple biological functions of Twist1, piecing together the whole delicate picture of Twist1's diversified pathological roles in different cancers and providing new perspectives to guide future research.

## INTRODUCTION

Twist1, a basic helix-loop-helix (bHLH) domain-containing transcription factor, was originally identified in Drosophila as an essential regulator during embryogenesis, particularly in mesoderm formation, specification, and differentiation [1, 2]. Drosophila embryos harboring Twist1 mutations fail to invaginate properly, resulting in embryos devoid of internal organs with a “Twisted” appearance [1, 3]. In humans, the Twist1 gene is located on 7q21.2 containing two exons and one intron [4]. Mutation of Twist1 in humans leads to Saethre-Chotzen syndrome, a disease of autosomal dominant inheritance characterized by manifestations such as craniosynostosis, ptosis, and hypertelorism [5–7]. Twist2 is another member of the Twist subfamily of bHLH protein in humans which shares great structural similarity with Twist1. Both Twist1 and Twist2 are key regulators in embryonic development and organogenesis. While a great number of studies have extensively demonstrated that Twist1 is implicated in tumor initiation, stemness, angiogenesis, dissemination, and chemoresistance in various carcinomas, sarcomas and hematological malignances, the biological functions of Twist2 in tumor are still highly controversial or unexplored [8–12]. Therefore, this review will focus mainly on Twist1.

**Figure 1:**
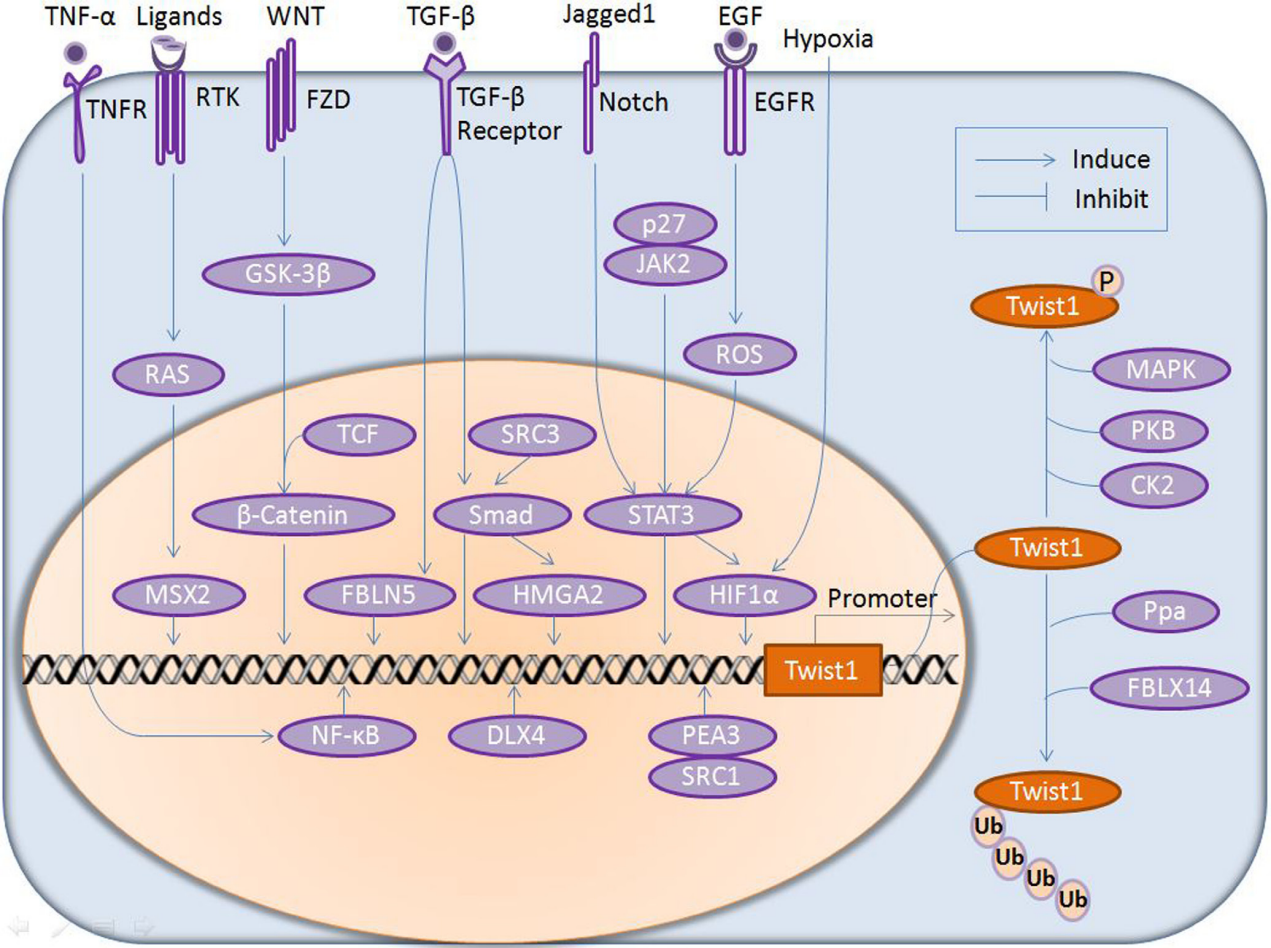
Modulation of Twist1 expression by upstream regulators at both gene and protein levels Manifold extracellular insults (such as TNF-α, RTK ligands, WNT, TGF-β, Jagged1, EGF signaling and hypoxia) are transduced into the cell *via* trans-membrane receptors (TNFR, RTK, FZD, TGF-β, Notch and EGFR) and intracellular mediators in the cytosol (MAPK, AKT) and nucleus (NF-κB, MSX2, β-catenin, FBLN5, Smad, HMGA2, STAT3 and HIF-1α), thus regulating Twist1 expression at the gene level and Twist1 stability at the protein level, respectively. P, phosphrylation; Ub, Ubiquitination; RTK, receptor tyrosine kinases; EGF, epidermal growth factors; FZD, frizzed; GSK-3β, glycogen synthase kinase 3β; TCF, transcription factor; JAK2, janus kinase 2.

**Figure 2:**
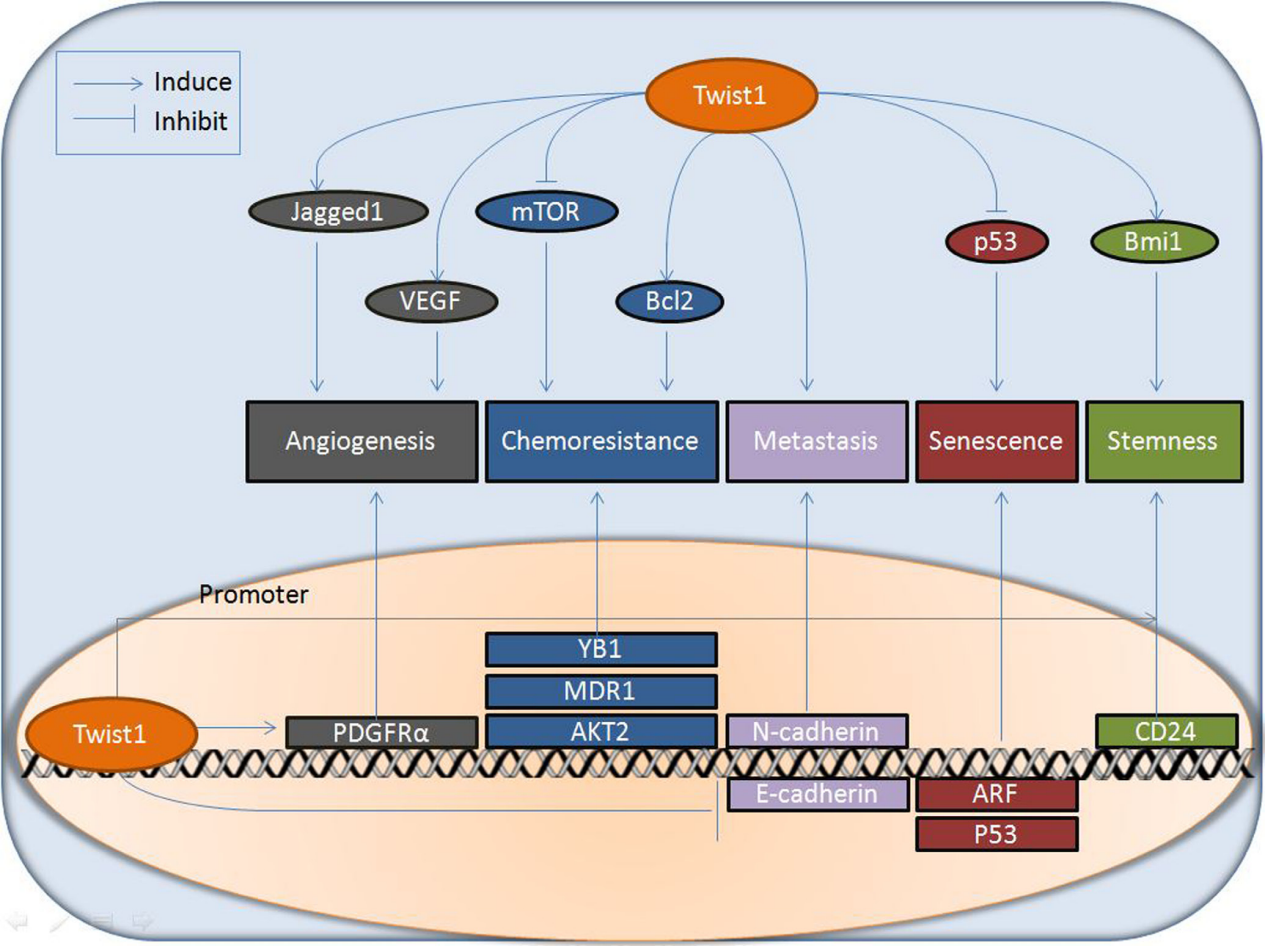
Downstream effectors involved in multiple functions of Twist1 in cancer Twist1 exerts its multiple biological effects (angiogenesis, chemo-resistance, metastasis, senescence, and stemness) *via* various downstream pathways, acting as a transcription factor regulating the expression of an array of target genes (such as PDGFRα, YB1, MDR1, AKT2, N-cadherin, E-cadherin, ARF, p53, and CD24) in the nucleus or modulating the function of effectors (e.g. Jagged1, VEGF, mTOR, Bcl2, p53, and Bmi1) at the protein level in the cytoplasm.

The physiological and pathological contributions of Twist1 to the development and progression of different diseases have been widely reviewed. However, limited reviews have systematically summarized the network of signaling pathways and the fundamental molecular basis underlying Twist1's multiple biological functions. Few reports have elaborated the independence and interdependency of Twist1's multiple distinct pathological functions. Therefore, in the current review, we will first outline the diverse pathological functions of Twist1 in various cancers, then delineate the intricate underlying molecular mechanisms and network of signaling pathways (as illustrated in Figures 1 and 2), based on which we will further summarize current therapeutic strategies in cancer treatment that target or modulate Twist1-involved signaling pathways (Table 1). More importantly, we put special emphasis on revealing the molecular basis of the correlation between Twist1's diversified biological functions, piecing together the whole delicate picture of Twist1's multiple roles in various cancers and providing new directions for future research.

**Table 1:** Emerging role of Twist1 in drug resistance

Involved molecular pathways		Drug resistance phenotypes	Cancer types
Twist1 promotes the expression of ABC transporters(e.g. MDR1)		Induction of MDR	Breast cancer [102, 105]
		Induction of resistance to anthracycline drugs	Bladder cancer[103, 104]
		Induction of oxaliplatin resistance	Colorectal cancer [37]
Activation of PI3K/AKT pathways		Induction of vincristine and paclitaxel resistance	Nasopharyngeal cancer; bladder cancer; prostate cancer; ovarian cancer[107, 108]
Twist1 induces AKT2 expression		Induction of paclitaxel resistance	Breast cancer [109]
Twist1 down-regulates Bcl2		Reversion of NF-κB induced chemo-resistance	Prostate cancer [110]
Twist1 increases YB-1 expression		Induction ofcsplatin and doxorubicin resistance	Bladder cancer[112]
Twist1 activates Jagged1/KLF4 pathway		Induction of cisplatin and cetuximab resistance	Head and neck cancer [101]
Mitogen-activated protein kinases	Down-regulation of Twist1 activates ERK pathway	Reversion of radio-resistance	Nasopharyngeal cancer [113]
	Twist1 increases p38 MAPK activity	Induction of cisplatin resistance	Pancreatic cancer [114]
	Twist1 silencing activates JNK pathway	Reversion of cisplatin resistance	Lung cancer [115]
Twist1 depletion inhibits mTOR and reduces Mcl1 expression		Reversion of cisplatin resistance	Lung cancer[116]
micro-RNAs	miR-186 reduces Twist1 expression	Reversion of cisplatin resistance	Ovarian cancer [117]
	Inhibition of miR-106a up-regulates Twist1 expression	Induction of gemcitabine resistance	Hepatocellular carcinoma[118]
	miR-181a suppresses Twist1 expression	Reversion of cisplatin resistance	Tongue squamous cell carcinoma [119]
	miR-23a increases Twist1 expression	Induction of cisplatin resistance	Tongue squamous cell carcinoma cancer[120]
Twist1 inhibits PI3K/AKT and down-regulates ET1/ETAR signaling		Reversion of cisplatin resistance	Osteosarcoma [121]
miR-33a reduces Twist1 expression		Induction of cisplatin resistance	Osteosarcoma[122]

## HYPERMETHYLATION AND OVER-EXPRESSION OF TWIST1 AS A PROGNOSTIC MARKER

The notion that Twist1 is involved in cancer pathology triggered extensive analysis of Twist1 promoter hypermethylation, mRNA expression and protein level in a wide variety of tumors. Twist1 promoter hypermethylation, one of the most important factors in the epigenetic reprogramming of Twist1, has been identified in cancers of different origins such as breast, bladder, gastric, colon and rectum, lung, ovary, and uterine cervix cancer [10]. It is noteworthy that the hypermethylation of Twist1 promoter is not necessarily related to Twist1 mRNA or protein expression. Twist1 over-expression is commonly observed in breast cancer [13–18], bladder cancer [19–21], gastric cancer [22, 23], hepatocellular carcinoma [24], esophageal squamous cell carcinoma [25, 26], nasopharyngeal carcinoma [27, 28], head and neck cancer (HNC) [29], glioblastoma [30, 31], and chronic myelogenous leukemia [32]. Consistent with its significance in cancer biology, Twist1 over-expression is related to high grade or invasive aggressive cancer with lymph node involvement or distant metastasis indicating therapeutic failure, recurrence, and inferior prognosis [8]. As a result, Twist1 might serve as a useful diagnostic or prognostic factor predicting poor outcome, which has already been illustrated in breast cancer [15], bladder cancer [33], cervical cancer [34], esophageal squamous cell carcinoma [35], HNC [36], colorectal cancer [37], hepatocellular cancer [38, 39], melanoma [40], nasopharyngeal cancer [27], and ovarian cancer [41, 42]. The strength of the evidence supporting this notion ranges from cell lines, animal models to patient tissues, however, to date Twist1 detection is still far from clinical application. There are many obstacles hampering the clinical use of Twist1 as a diagnostic or prognostic marker, such as the lack of efficient, precise and economic detection methods and the unknown basal level of Twist1 expression in normal tissues.

## UPSTREAM REGULATORS MODULATING TWIST1 FROM THE TRANSCRIPTIONAL TO POST-TRANSLATIONAL LEVEL

At the gene level, the expression of *Twist1* is modulated by an array of different upstream regulators *via* multiple pathways depending on the cancer type and tissue context (Figure 1). Induction of these pathways results in activation of the *Twist1* promoter by a variety of transcription factors in the nucleus. Firstly, signal transducer and activator of transcription 3 (STAT3) is implicated in Twist1 regulation by the observation that Twist1 expression is ablated upon STAT-3 knockdown in breast tumors in immunocompetent mice [43]. A subsequent study further confirmed that STAT3 directly binds to the second proximal STAT-3-binding site on the human *Twist1* promoter and activates its transcriptional activity in human breast cancer cell lines [44]. This STAT-3/Twist1 axis has also been verified in hepatocellular carcinoma cells [45] and the Notch1/STAT3/Twist1 signaling axis has been identified in gastric cancer [46]. In addition to binding to *Twist1* promoter directly, STAT3 is also demonstrated to regulate the transcription of *Twist1* indirectly through HIF-1 stabilization in prostate cancer cells [47]. HIF-1, in turn, regulates expression of *Twist1* by directly binding to the hypoxia-response element (HRE) in the *Twist1* proximal promoter [36].

NF-κB is another transcription factor that can directly reprogram the gene expression of *Twist1*. In early Drosophila embryos, the NF-κB-like transcription factor dorsal (dl) activates *Twist1* expression through binding to dl-binding sites in the *Twist1* promoter [48]. This pattern of *Twist1* regulation was later proven to be evolutionarily conserved in vertebrates by the observation that mammalian *Twist1* expression is induced by TNF-α in an NF-κB dependent manner in immortalized fibroblasts derived from p65−/− mice [49]. In mammary epithelial cells, NF-κB and Twist1 expression are positively related and a functional NF-κB-binding site has been identified in the *Twist1* promoter, further confirming the transcriptional regulation of *Twist1* by NF-κB [50].

The steroid receptor co-activators (SRC, also known as nuclear receptor co-activators), in particular SRC1 and SRC3, also participate in tuning *Twist1* expression. SRC3 can directly enhance Smad2 expression and subsequently increase *Twist1* transcription. Knocking down SRC3 reduces the expression of Smad and Twist1 *in vitro* and suppresses tumor metastasis *in vivo* [51]. Alternatively, Qin *et al*. revealed that SRC1 is able to activate *Twist1* transcription by physically interacting with the transcription factor polyoma enhancer activator 3 (PEA3) at the proximal *Twist1* promoter by comparing SRC-1 wild-type (WT) with knockout (KO) cell lines [52]. PEA3 is also proposed to be involved in the *Twist1* transcription regulation through the Wnt/β-catenin axis in mouse mammary cells [53]. The distal-less homeobox gene 4 (DLX4), a member of the DLX family widely expressed in various cancers, has also been shown to bind to regulatory regions of the *Twist1* gene and enhances tumor migration, invasion, and metastasis in cell models and tumor tissues [54].

Moreover, Ras and TGF-β signaling pathways regulate *Twist1* expression at both the transcriptional and protein levels. Specifically, the Msh homeobox protein (MSX2) is a downstream target of the Ras signaling pathway and is suggested to induce *Twist1* expression in human pancreatic cancer cells [55]. TGF-β/Smad signaling induces high mobility group A2 (HMGA2) transcription and the latter can induce expression of Twist1 by directly associating with A:T-rich sequences and promoting transcription from the *Twist1* promoter in mammary epithelial cells [56]. TGF-β can also induce fibulin 5 (FBLN5), which promotes tumor invasion and epithelial-mesenchymal transition (EMT) by elevating *Twist1* transcription and reducing E-cadherin expression, although the precise mechanism by which FBLN5 regulates *Twist1* transcription remains elusive [57].

Post-translational modifications are also crucial for the function of Twist1 protein in the cytosol. Phosphorylation and ubiquitination are the most important post-translational modifications that affect Twist1 function by regulating the stability of Twist1 protein [58]. Both Ras activation and TGF-β treatment are capable of activating the mitogen-activated protein kinases (MAPK) pathway, which significantly increases Ser68 phosphorylation of Twist1 and prevents Twist1 from undergoing E3-mediated ubiquitination and degradation without altering Twist1 mRNA expression in breast cancer cells [59]. Activation of PKB (AKT1) leads to a marked increase in Twist1 phosphorylation at Ser42 in the nucleus. Subsequent study further confirms that AKT1 physically associates with Twist1 and phosphorylation of Twist1 by AKT1 is required for Twist1 ubiquitination and degradation [60]. While AKT2 primarily phosphorylates Twist1 at Ser42, AKT1 phosphorylates Twist1 at Ser42 and Thr121 *in vitro* and at Ser42 *in vivo* [61]. Casein kinase 2 (CK2) interacts with Twist1 directly and phosphorylates Twist1 at Ser18 and Ser20, resulting in prolonged stability of Twist1 and enhancing the motility of HNC cells [62].

In contrast to increasing Twist1 stability, several publications have reported that the F-box protein Ppa, as well as its human homologue FBXL14, can mediate and induce the poly-ubiquitination and degradation of Twist1 protein in embryos and in HNC cells, respectively [63, 64].

Post-translational acetylation can also affect the function of the Twist1 protein. Evidence from basal-like breast cancer (BLBC) cells demonstrated that Twist1 can be diacetylated at K73 and K76 by Tip60. This diacetylation is critical for Twist-BRD4 interaction and the subsequent formation of activated Twist/BRD4/P-TEFb/RNA-Pol II complex at the WNT5A promoter and enhancer. The Twist-BRD4-WNT5a axis is crucial for the tumorigenicity of basal-like breast cancer both *in vitro* and *in vivo*. Disrupting the interaction of BRD4 with Twist1 by using BET-specific inhibitors (such as JQ1) may be a novel approach to indirectly suppress Twist1 function and provide a potential new target for the treatment of basal-like breast cancer [65].

## DOWNSTREAM EFFECTORS INVOLVED IN MULTIPLE FUNCTIONS OF TWIST1

Twist1 can exert multiple biological effects *via* various downstream pathways as either a transcriptional factor regulating the expression of an array of target genes or a functional modulator at the protein level (Figure 2). The most critical pathological function of Twist1 in cancer is facilitating tumor invasion and metastasis by promoting EMT [13]. Many studies have shown that Twist1 is also involved in other facets of tumor invasion and metastasis, such as the formation of invadopodia [66], intravascular migration, extravasation [67], and vasculogenic mimicry (VM) formation [68]. Furthermore, growing evidence indicates that Twist1 also plays a crucial role in supporting tumor initiation by evading p53 induced cell senescence and apoptosis, the well-known program to counter cell transformation [69–71]. In addition, evidence has shown that Twist1 can confer cancer cells stemness properties [72–73]. Moreover, the cross-talk between Twist1 and tumor angiogenesis has also attracted great attention [74]. Recently, many studies have also shed light on the connection between Twist1 and chemoresistance. At the same time, tremendous efforts have been made to modulate Twist1 as a therapeutic target for cancer treatment [10]. The multiple biological functions of Twist1 are elaborated in the following sections:

### Contribution of Twist1 to tumor invasion and metastasis

Cancer metastasis consists of a sequence of distinct yet closely related steps: EMT, local invasion, intravasation, transit in blood or lymphatic circulation, extravasation, micro-metastasis, and colonization [75]. Twist1 has been well established as one of the master regulators of the EMT process. Additionally, extensive studies have demonstrated that Twist1 is also involved in other steps of the tumor invasion and metastasis process such as the formation of invadopodia [66], intravascular migration, extravasation [67] and vasculogenic mimicry (VM) formation [68, 76].

About a decade ago, Yang *et al*. first established the link between Twist1, EMT, and tumor metastasis by comparing the gene profile of different mouse tumor cell lines isolated from the same breast cancer [13]. Since then, the relationship between Twist1-induced EMT and cancer metastasis has been verified in a broad range of tumor types including hepatocellular carcinoma (HCC) [24], prostate cancer [77], gastric cancer [22], esophageal squamous cell carcinoma [25, 35], bladder cancer [19], pancreatic cancer [55], gliomas [30, 31], nasopharyngeal carcinoma [28], HNC [29], and epithelial ovarian carcinoma [78].

Although the up-stream regulators of Twist1 vary largely from one cancer to another during EMT, the down-stream effectors always involve the up-regulation of N-cadherin and/or down-regulation of E-cadherin. Twist1 has been demonstrated to induce N-cadherin at the mRNA level through the E-box cis-element located within the first intron of the N-cadherin gene in prostate cancer [79]. Furthermore, Twist1 can bind directly to the E-cadherin promoter, down-regulate promoter activity, and repress E-cadherin gene expression [80]. Further study reveals in detail that Twist1 interacts with several components of the Mi2/nucleosome remodeling and deacetylase (Mi2/NuRD) complex (MTA2, RbAp46, Mi2, HDAC2) and recruits them to the proximal regions of the E-cadherin promoter for transcriptional repression [81].

In addition to these cardinal EMT pathways, Twist1 also regulates other aspects of the tumor metastasis process *via* different signaling pathways. By using real-time intravital imaging of human tumor cells transplanted into transparent zebrafish, Stoletov *et al*. demonstrated that the expression of Twist1 in tumor cells increases their intravascular migration and extravasation through the vessel wall [67]. In addition, Twist1 promotes invadopodia formation *via* up-regulation of platelet-derived growth factor receptor (PDGFR) expression and activity [82]. Sun *et al*. discovered that Twist1 is frequently over-expressed in VM-positive HCCs, suggesting that Twist1 expression is likely to be associated with VM formation. This group further confirmed that over-expression of Twist1 significantly enhanced cell motility, invasiveness, and VM formation while Twist1 depletion substantially reduced cell migration, invasion, and VM formation [68].

Some microRNAs can also represent the target of Twist1 and mediate Twist1-induced EMT [83]. By binding to the putative promoter of miR-10b, Twist1 can induce the expression of miR-10b which inhibits the translation of homeobox D10, leading to the induction of pro-metastatic gene RHOC in breast cancer cells and a mouse model [84]. Additionally, Twist1 can suppress the expression of let-7i, resulting in the activation of RAC1 and enabling mesenchymal-mode movement in three-dimensional environments [85].

### Twist1 as an oncoprotein promoting tumor initiation by evading senescence and apoptosis

Oncogenic insults usually induce p53 and/or retinoblastoma (Rb) expression and result in cell apoptosis or senescence, which are well-known defensive barriers against cell transformation and tumor initiation [86, 87]. Twist1 protein has been shown to override this safe-guard program to evade oncogene-induced senescence and apoptosis. Inactivation of Twist1 leads to the promotion of cellular senescence and cell growth arrest. In contrast, over-expression of Twist1 results in suppression of cellular senescence in response to genotoxic damage and promotion of cell proliferation with DNA damage accumulation [70]. Vichalkovski *et al*. demonstrated that PKB/AKT2 can phosphorylate Twist1 at Ser42 and inhibit p53 activity in response to DNA damage and this post-translational modification ensures functional activation of Twist1 after the promotion of survival during carcinogenesis [61]. Furthermore, by applying a functional screen for cDNAs that counteract the pro-apoptotic effects of the MYC oncogene, Maestro's group discovered that Twist1 can bypass and inhibit p53-dependent cell death [88]. They proposed that Twist1 is capable of reducing expression of the ARF tumor suppressor and affecting p53 indirectly through modulation of the ARF/MDM2/p53 pathway. Consistent with this proposal, Kwok *et al*. demonstrated that Twist1-mediated cellular senescence was regulated through its negative effect on p14 (ARF) and subsequent suppression of MDM2/p53 and Chk1/2 DNA damage response pathways in prostate epithelial cells [70]. Similar results were observed by Valsesia *et al*. who found that Twist1 over-expression is responsible for inhibition of the ARF/p53 pathway involved in the Myc-dependent apoptotic response in neuroblastomas [71]. In addition to indirectly regulating p53 *via* ARF/MDM2, Paccinin *et al.* also demonstrated that Twist1 directly binds the p53 C-terminus through the Twist1 box which hinders the post-translational modification of p53 and facilitates its MDM2-mediated degradation [89]. Aside from regulating p53 at the post-transcription level, Twist1 was also shown to physically interact with HOXA-5 and negatively regulate p53 gene expression at the transcriptional level [90].

Conversely, Pinho *et al*. discovered that p53(−/−) pancreatic epithelial cells undergo EMT and express high levels of vimentin and of the transcriptional regulators Snai1, Snai2, Twist1, Zeb1 and Zeb2, implying that p53 inactivation in turn may promote Twist1 expression [91]. This study raised the possibility of mutual reciprocal regulation between Twist1 and p53, filling in the gap between p53 inactivation in tumor initiation and Twist1-induced tumor metastasis. This notion is supported by the study of Ansieau's group who demonstrated that Twist1 overrides oncogene-induced premature senescence and simultaneously induces complete EMT, suggesting that some metastatic capabilities of cancer cells can be acquired during malignant conversion as a side effect of the inactivation of primary gatekeeper mechanisms [92]. However, Beck *et al*. established that Twist1 controls tumor initiation in a p53-dependent and -independent manner in the absence of EMT induction, suggesting Twist1-induced tumor initiation and EMT are not necessarily functionally linked [72]. This concept is discussed further in the perspectives and future directions section below.

### Twist1 in a circuit of cancer stemness

Multiple lines of evidences have demonstrated the connection between EMT and cancer cell stemness [93]. One study reported that induction of EMT in immortalized human mammary epithelial cells (HMLEs) results in the expression of stem-cell markers and increased ability to form mammospheres, a property associated with mammary epithelial stem cells [94]. The same group further demonstrated that the EMT-derived cells are similar to mesenchymal stem cells (MSCs) in gene expression, multi-lineage differentiation, and migration ability. The functional connection between EMT and cancer stemness is molecularly mediated by the interdependency between Twist1 and Bmi-1 [95]. Bmi-1 is a polycomb-group protein that maintains self-renewal and stemness properties, which can be transcriptionally regulated by Twist1 *via* direct binding to the element on the Bmi-1 promoter. Bmi-1 knockdown leads to reversion of EMT while over-expression of Bmi-1 induces EMT, suggesting that Bmi-1 is critical mediator for Twist1-induced EMT. Conversely, silencing of Twist1 in Bmi1-overexpressing cells abolishes both EMT and stem-like properties. As a result, the interdependency between Twist1 and Bmi-1 provides the molecular connection between Twist1-induced EMT and stemness [73]. On the contrary, it is noteworthy that Twist1 can also promote the acquisition of stemness properties independent of EMT, as demonstrated by Vesuna *et al*. who established that the over-expression of Twist1 in breast cells can transcriptionally regulate CD24 expression and promote the generation of a breast cancer stem cell phenotype characterized by the high expression of CD44, and little or no expression of CD24 without inducing EMT [96]. In agreement with this study, Beck *et al.* also demonstrated that a low level of Twist1, which is insufficient to induce EMT, is able to endow skin tumor stemness [72]. Similarly, Schmidt *et al*. proposed that transient activation of Twist1, which is insufficient to induce EMT, is able to promote stem-cell-like properties such as mammosphere formation in human mammary epithelial cells. Persistent Twist1 could induce EMT but inhibits stem-cell-like properties and stemness only emerges and stably persists following Twist1 deactivation, implying the mutually exclusive nature of Twist1-induced EMT and stemness (see more detail in the perspectives and future directions section below) [97]. Taken together, this evidence suggests that it is the target genes down-stream of Twist1 (for example: CD24), rather than EMT, that confers cancer stem cell properties [98].

### The association of Twist1 with tumor angiogenesis

Angiogenesis is a normal process that is transiently turned on under physiologic conditions such as female reproductive cycling and wound healing. In contrast, during tumor progression, angiogenesis is continually activated to help sustain expanding neoplastic growth [86]. Accumulating evidence has shown that Twist1 is positively associated with tumor angiogenesis [74]. Over-expression of Twist1 in breast cancer increases vascular endothelial growth factor (VEGF) expression and induces angiogenesis *in vivo* [99]. In agreement, Twist1 expression is positively correlated with up-regulation of VEGF in hepatocellular carcinoma cells and HCC specimens with positive Twist1 expression have a higher micro-vessel density than those without Twist1 expression [38]. Moreover, Twist1 is required for thrombin-induced angiogenesis and thrombin up-regulates Twist1, thus promoting endothelial cell migration, matrigel tubule formation, and tumor angiogenesis [100]. The same study shows that Twist1 mediates the thrombin induced up-regulation of angiogenesis growth factors and receptor proteins such as VEGF, GRO-α, KDR, Ang-2, MMP-1, and CD31 in both human breast cancer and murine melanoma cell lines. In addition to regulating vascular growth factors/receptors and activating endothelial cells in existing vessels, Twist1 can also enhance angiogenesis by inducing trans-differentiation of tumor cells into endothelial cells [101]. Twist1 can induce Jagged1 expression and subsequently activate KLF4, leading to endothelial differentiation in HNC cells. Interestingly, Bmi-1 is identified as another downstream effector of Twist1/Jagged-1 and this Twist1/Jagged-1/Bmi-1 axis confers stemness properties in HNC cells, indicating the molecular association between Twist1-induced angiogenesis and stemness [101].

### Emerging role of Twist1 in drug resistance

Chemo-resistance is one of the biggest obstacles to the successful treatment of many cancers. Recently, emerging evidences has demonstrated that Twist1 can confer chemo-resistance in various cancer cell types. As summarized in Table 1, much effort has been made to explore the role of Twist1 in drug resistance.

Generally, the development of multidrug resistance is associated with increased expression of several ATP binding cassette transporters (ABC transporters) including ABCC1, ABCC3, ABCC4, ABCC5, and ABCC10. Saxena *et al*. found that Twist1 binds directly to the E-box elements of ABC transporters, identifying a molecular connection between Twist1 and multiple drug resistance [102]. Moreover, P-glycoprotein (P-gp, also known as MDR1) is a well-known member of the MDR/TAP subfamily of ABC transporters that pump many foreign substances (chemo-therapeutics) out of cells. Twist1 is co-expressed with P-gp in human bladder cancer cells and knockdown of Twist1 significantly sensitizes bladder cancer cells to anthracycline drugs *via* inhibiting P-gp expression [103]. The same group further demonstrated that DAB2IP can inhibit the phosphorylation and transactivation of STAT3, subsequently suppressing the expression of Twist1 and its target gene P-gp. This DAB2IP/STAT3/Twist1/P-gp axis is crucial for chemo-resistance to the anthracycline drugs (pirarubicin) and tumor re-growth of bladder cancer cells [104]. In agreement with this study, Li *et al*. demonstrated that Twist1-mediated EMT results in multidrug resistance and Twist1 depletion improves the efficacy of doxorubicin partially by suppression of drug-induced P-gp expression in breast cancer cells [105]. In addition, Deng *et al*. recently demonstrated that Twist1 and P-gp are also expressed correlatively in colorectal cancer cells (CRC) and confer CRC chemo-resistance to oxaliplatin [37]. Controversially, a recent study by Kong's group suggests that the expression of ABC transporters (MDR1 and BCRP) is dispensable for the chemo-resistance mediated by Twist1 [106]. This seeming discrepancy might be explained by the use of cell lines with artificially elevated Hedgehog signaling pathway by Kong *et al*., which could enhance MDR1 and BCRP transcription, thus compensating for or even reversing the potential down-regulation of MDR1 and BCRP upon Twist1 knockdown.

In nasopharyngeal carcinoma, Twist1 is identified to play a central role in acquired resistance to paclitaxel. Up-regulation of Twist1 is associated with cellular resistance to microtubule-targeting anticancer drugs (vincristine and paclitaxel) but not to other drugs in nasopharyngeal, bladder, ovarian, and prostate cancer cells [107]. A subsequent mechanistic study by the same group suggests that Twist1-mediated paclitaxel resistance may be regulated through its positive involvement with the PI3K/AKT pathway [108]. In addition to the AKT pathway, Twist1 also positively regulates AKT2 expression by binding to E-box elements on the AKT2 promoter in breast cancer cells. AKT2 functions as a down-stream target of Twist1 and mediates Twist1-induced migration, invasion, and paclitaxel resistance [109]. In prostate carcinoma cells, Pham *et al*. demonstrated that Twist1 serves as a down-stream target of NF-κB, mediating NF-κB-induced chemo-resistance and TNF-α induced programmed cell death. Further detailed mechanistic study revealed that the protective activity of Twist1 seems to halt programmed cell death by controlling inhibitory Bcl-2 phosphorylation independently of interference with cytotoxic JNK, p53, and p19 (ARF) signaling [110]. In pancreatic cancer, deletion of mouse Twist1 did not halt the invasion, systemic dissemination or metastasis of pancreatic ductal adenocarcinoma (PDAC), but sensitized tumors to gemcitabine *in vivo* [111]. In bladder cancer, Twist1 regulates Y-box-binding protein-1 (YB1) expression and both Twist1 and YB1 are involved in cell growth, invasion, motility, and resistance to cisplatin and doxorubicin, but not to 5-fluorouracil (5-FU) [112]. As mentioned above, Twist1 can induce Jagged-1 expression and subsequently activate KLF4, not only inducing angiogenesis, but also conferring cisplatin and cetuximab resistance in HNC cells [101]. Of note, mitogen-activated protein kinases (ERK, JNK, and p38) are important down-stream mediators of Twist1-induced drug-resistance, but the specific effector varies from cancer to cancer. In nasopharyngeal carcinoma cells, down-regulation of Twist1 increases radio-sensitivity by inducing activation of the ERK pathway, but not the p-38 or JNK pathway [113]. However in pancreatic cancer cells, Twist1 promotes invasion and cisplatin resistance by inducing GDF15 expression by increasing p38 MAPK activity [114]. In non-small cell lung cancer (NSCLC), Zhuo *et al*. demonstrated that Twist1 silencing significantly sensitizes NSCLC cells to cisplatin by activating the JNK/mitochondrial pathway but not the ERK and p38 pathways [115]. Interestingly, Jin *et al*. proposed that Twist1 depletion sensitized NSCLC cells to cisplatin by stimulating AMPK-induced mTOR inhibition and subsequent reduction in Mcl-1 protein [116]. It is noteworthy that both groups used the same NSCLC cell line (A549) and drug (cisplatin) but obtained different results, implying that Twist1 might be able to mediate chemo-resistance through multiple down-stream pathways even in the same cancer type.

Recently, multiple studies suggest that various micro-RNAs are involved in chemo-resistance in a Twist1-dependent manner. Cisplatin-resistant ovarian cancer cell lines exhibit decreased miR-186 expression and increased Twist1 expression while the introduction of miR-186 can reverse drug resistance through Twist1 down-regulation [117]. Wang et al. demonstrated that PDGF-D markedly inhibited miR-106a expression and subsequently up-regulated Twist1 expression in gemcitabine-resistant hepatoma cells [118]. In tongue squamous cell carcinoma, Liu et al. found that miR-181a could reverse cisplatin resistance by directly targeting and repressing Twist1 expression [119]. In addition, miR-23a promoted cisplatin chemo-resistance and protected cisplatin-induced apoptosis through induction of Twist1 expression *via* a JNK-dependent mechanism in tongue squamous cell carcinoma cells [120].

Interestingly, down-regulation, rather than up-regulation of Twist1 is implicated in chemo-resistance in osteosarcoma. Zhou et al. demonstrated that Twist1 markedly decreases osteosarcoma cell survival following cisplatin treatment partially by down-regulating the endothelin-1 (ET1)/endothelin A receptor (ETAR) signaling *via* inhibition of the PI3K/AKT pathway [121]. Consistently, a subsequent study found that miR-33a promotes osteosarcoma cell resistance to cisplatin by down-regulating Twist1. Inhibition of miR-33a up-regulates Twist1 expression and enhances cisplatin-induced apoptosis [122].

## PERSPECTIVES AND FUTURE DIRECTIONS

Taken together, multiple studies show that Twist1 is a transcription factor that is over-expressed in a wide variety of carcinomas, and is implicated in many aspects of the carcinogenesis process including tumor initiation, stemness, angiogenesis, invasion, metastasis, and drug resistance. Among these varied functions, the contribution of Twist1 to EMT is the most thoroughly explored and extensively verified in various cancers. The involvement of Twist1 in tumor initiation, stemness, angiogenesis, and drug resistance and the underlying mechanisms remain largely undefined. Further detailed studies exploring how Twist1 participates in these processes will provide more comprehensive information about the crucial pathological functions of Twist1 in cancer. Moreover, instead of studying these seemingly independent pathological effects of Twist1 separately, there is growing interest in exploring the correlation and interdependency of these multiple biological functions. For example, Ansieau's group discovered that Twist1 overrides oncogene-induced premature senescence and simultaneously induces complete EMT, indicating that Twist1-induced early escape from gatekeeper protection and the acquisition of invasive features are directly linked [69]. However, Beck *et al*. established that low levels of Twist1 control tumor initiation in both a p53-dependent and -independent manner without inducing EMT, suggesting that Twist1-induced tumor initiation and EMT are not necessarily functionally related [72]. This apparent inconsistency raises the notion that Twist1 function might be dictated by its expression level, namely, a low level of Twist1 promotes tumor initiation while a relatively higher level of Twist1 expression is a prerequisite for EMT induction. In agreement with this theory, Beck *et al*. demonstrated that a low level of Twist1, which is insufficient to induce EMT, is able to confer skin tumor stemness [72]. Continued efforts should be made to further explore the correlation between Twist1 expression level and its biological effects.

Twist1-induced EMT and stemness are suggested to be functionally associated by the molecular connection between Twist1 and Bmi-1 [73]. In contrast, Twist1 can also promote the acquisition of stemness properties in the absence of EMT induction. Vesuna *et al.* established that the over-expression of Twist1 in breast cells can transcriptionally regulate CD24 expression and promote the generation of a breast cancer stem cell phenotype independent of EMT [96]. This discrepancy may be explained by Schmidt's observation that transient activation of Twist1, at a level insufficient to induce EMT, is able to promote stem-cell-like properties. Persistent Twist1 can induce EMT but inhibit stem-cell-like properties and stemness only emerges and stably persists following Twist1 deactivation, implying the mutually exclusive nature of Twist1-induced EMT and stemness [97]. As a result, in addition to regulating tumor biology in an expression level-dependent manner, the temporal regulation of Twist1 is also important for its distinctive biological functions. The “spatiotemporal” regulation of EMT by Twist1 was suggested by Tsai et al. who proposed that different Twist1 expression levels at different times in different places dictate EMT, tumor dissemination, proliferation and the formation of metastases [123]. Still, little is known about the temporal regulation of Twist1 and this field could be a promising direction for future research.

Twist1-induced angiogenesis and stemness are also functionally connected. Twist1 can induce Jagged-1 expression and subsequently activate KLF-4, leading to endothelial differentiation in HNC cells. Interestingly, Bmi-1 is identified as another downstream effector of Twist1/Jagged-1 and this Twist1/Jagged-1/Bmi-1 axis confers stemness properties in HNC cells, indicating the molecular connection between Twist1-induced angiogenesis and stemness [101]. Cancer stemness is well-established to be closely related to drug resistance [124, 125]. However, the detailed signaling pathways linking Twist1-induced drug resistance and cancer cell stemness remain elusive.

In addition to pursuing these many promising fields of Twist1 bench research, several areas can be further explored on the bed side. To date, little is known about the basal level of Twist1 in different tissues in healthy individuals and there is no efficient method for Twist1 detection. Quantitative information about the genetic mutation, amplification, and deletion of Twist1 is also lacking.

In summary, based on the significance of Twist1 in various hallmark properties of cancer, detailed studies are needed to delineate and unveil the underlying mechanisms of Twist1's multiple pathological functions. A comprehensive understating of Twist1's diversified roles in cancer biology will lay the foundation for further modulation of Twist1 as a diagnostic indicator, prognostic marker, and a therapeutic target for cancer treatment in the clinic.
